# Qian Yang Yu Yin Granule Improves Renal Injury of Hypertension by Regulating Metabolic Reprogramming Mediated by HIF-1α/PKM2 Positive Feedback Loop

**DOI:** 10.3389/fphar.2021.667433

**Published:** 2021-06-07

**Authors:** Lichao Qian, Shuai Ren, Zhongchi Xu, Yawei Zheng, Lihua Wu, Ying Yang, Yixuan Wang, Jie Li, Shihai Yan, Zhuyuan Fang

**Affiliations:** Jiangsu Province Hospital of Chinese Medicine, Affiliated Hospital of Nanjing University of Chinese Medicine, Nanjing, China

**Keywords:** hypertensive nephropathy, metabolic reprogramming, HIF-1α, PKM2, Qian Yang Yu Yin granule

## Abstract

Protection against hypoxia injury is an important therapeutic strategy for treating hypertensive nephropathy. In this study, the effects of Qian Yang Yu Yin granule (QYYY) on spontaneously hypertensive rats fed with high salt diet and HEK293T cells exposed to hypoxia were investigated. After eight weeks’ treatment of QYYY, blood pressure, serum creatinine, serum cystatin C, blood urea nitrogen, urinary β2-microglobulin, urinary N-acetyl-β-glucosaminidase, and urinary microalbumin were assessed. The changes of hypoxia-inducible factor-1α (HIF-1α), pyruvate kinase M2 (PKM2), glucose transport 1 (GLUT1), lactate dehydrogenase A (LDH-A), connective tissue growth factor (CTGF), transforming growth factor-β1 (TGF-β1), ATP, lactate, pyruvate, and pathology were also assessed *in vivo*. HEK293T cells pre-treated with QYYY and/or HIF-1α over expressing cells were cultured in a three gas hypoxic incubator chamber (5% CO_2_, 1% O_2_, 94% N_2_) for 12 h and then the expressions of HIF-1α, PKM2, GLUT1, LDH-A, CTGF, TGF-β1, ATP, lactate, and pyruvate were detected. Our results showed that QYYY promoted the indicators of renal inflammation and fibrosis mediated by HIF-1α/PKM2 positive feedback loop *in vivo* and *vitro*. Our findings indicated that QYYY treated hypertensive nephropathy by regulating metabolic reprogramming mediated by HIF-1α/PKM2 positive feedback loop.

## Introduction

Hypertension is a commonly observed cardiovascular disease and kidney damage is one of the major complications found in patients with hypertension. Hypertension is one of the important causes of chronic kidney disease. Statistics from the American Nephrology Association in 2009 showed that about 28% of patients with end-stage kidney disease were induced by hypertension (Mahmoodi et al., 2012). Traditional Chinese medicine (TCM) has its unique curative effects in the treatment of hypertensive renal damage, but the mechanism of TCM in the treatment of hypertensive renal damage is insufficient (Guo et al., 2019). Under the guidance of the theory of “blood stasis and heat” in TCM, Qian Yang Yu Yin granule (QYYY) was created and applied as hospital preparation and has been widely used in clinical practice for more than 20 years in Jiangsu Province Hospital of Chinese Medicine. It is safe and effective in treating hypertensive kidney injury. Previous studies of our project group showed that QYYY had antioxidant, anti-inflammatory and anti fibrosis effects (Chen et al., 2013; Wang et al., 2014; Yan D et al., 2014; Ding et al., 2015; Yan et al., 2018; Liu et al., 2019; Zhang et al., 2020).

Metabolic reprogramming refers to the change of cell metabolic mode. In normal cells, when oxygen is sufficient, cells primarily obtain energy through the process of oxidative phosphorylation. However, under hypoxia, cells obtain energy through glycolysis, pentose phosphate and other pathways. This switch of cell energy acquisition mode has been termed as metabolic reprogramming (Vander et al., 2009; Krisher and Prather, 2012). Kidney is an organ that requires high-energy consumption. Therefore, the normal and optimal energy metabolism system serves as an important biochemical basis to maintain the specific structure and physiological functions of the kidney. A number of previous studies have reported that alteration in renal cell metabolism from oxidative phosphorylation to glycolysis is the main feature of cell activation during renal fibrosis and inhibition of renal cell glycolysis can significantly reduce renal fibrosis (Ding et al., 2017). Moreover, gene and protein analyses have showed that the expression of glycolytic enzyme was significantly up-regulated in renal interstitial fibroblasts treated with unilateral ureteral obstruction (UUO) or transforming growth factor-β1 (TGF-β1). In nephropathy caused by UUO or renal interstitial fibroblasts treated with TGF-β1, the aerobic glycolysis flux was increased with glucose uptake and lactate production and it was reported to be positively correlated with the fibrosis process. Glycolysis inhibitors can be used as a potential anti fibrosis strategy.

Protection against hypoxia injury is an important therapeutic strategy for treating hypertensive nephropathy (Watson et al., 2019). Hypoxia inducible factor-1α (HIF-1α) is a special protein distributed in mammalian cells. Knockout or inhibition of HIF-1α effectively improved hypertensive renal injury (Huang et al., 2019). In renal injury model, HIF-1α mediated gene expression in renal medulla such as glucose transporter 1 (GLUT1), pyruvate kinase M2 (PKM2), connective tissue growth factor (CTGF), TGF-β1, and other related genes were increased. A related study showed that long-term over-expression of HIF-1α was a pathogenic factor leading to chronic kidney injury and stimulating the expression of HIF-1α in cells induced renal injury, hypertension and disease progression (Armutcu et al., 2019). Moreover, a related study found that the level of serum HIF-1α may reflect the degree of damage of chronic glomerulonephritis and actively participate in the occurrence and development of chronic glomerulonephritis (Dallatu et al., 2014). It has been reported that an increase of HIF-1α level could participate in the formation of proteinuria, promote metabolic reprogramming and renal fibrosis, and thereby aggravate the progressive deterioration of renal function. Our previous study found that HIF-1α was highly expressed in hypertensive renal injury model (Wu, 2020). Therefore, we speculated that HIF-1α is a key target of metabolic reprogramming of renal cell in hypertensive renal injury model.

A large number of studies have confirmed that HIF-1α is closely related to PKM2 (Luo et al., 2011). For instance, Hasan D found that the antisense chain of the first intron of PKM gene contained hypoxia response element (HRE), and proved that HIF-1α significantly promoted the expression of PKM2, which also formed a positive feedback mechanism at the gene level and played a key role in cell metabolic reprogramming (Hasan et al., 2018). In another study, Chai Xin Xin used shikonin, a specific inhibitor of PKM2, to down-regulate the expression of PKM2. They found that the down-regulation of PKM2 in breast cancer cells caused the down-regulation of HIF-1α (Chai et al., 2019). Therefore, we speculated that HIF-1α/PKM2 positive feedback is the key pathway of metabolic reprogramming of renal cell in hypertension.

## Materials and Methods

### Preparation of Qian Yang Yu Yin

QYYY (Batch No. Z20100007) was obtained from Jiangsu Province of Chinese Medicine. It is made up of *Cyathula officinalis* K. C. Kuan [Amaranthaceae; cyathulae radix], *Scrophularia ningpoensis* Hemsl [Scrophulariaceae; scrophulariae radix], *Bidens pilosa* L. [Asteraceae; *Bidens bipinnata* L.], *Cornus officinalis* Siebold and Zucc [Cornaceae; corni fructus], *Alisma plantago-aquatica* subsp. orientale (Sam.) Sam. [Alismataceae; alismatis rhizoma] and *Reynoutria multiflora* (Thunb.) Moldenke [Polygonaceae; polygoni multiflori radix] (Rivera et al., 2014). Detailed information on the components of QYYY is listed in Table 1. The preparing process of QYYY was as below: six ingredients were mixed together. Then the mixture was decocted with 12 times of the amount of water twice, for 1 h each time. The supernatant obtained by high-speed centrifugation was concentrated to a relative density of 1.2 g/ml at 60°C. After stevioside was added to a concentration of 1% (weight/volume), the concentrated supernatant was mixed with dextrin and granulated in a fluidized bed granulator. The obtained particles were dried at 60°C for 1 h and then stored at 4°C (Ding et al., 2015). High performance liquid chromatogram (HPLC) was established to determine the active components in QYYY and 2,3,5,4′-tetra-hydroxystilbene-2-O-β-Dglucopyranoside (2,3,5,4-TDG), morroniside, harpgide, ecdysterone, and hyperin were detected. The quality evaluation of QYYY is shown in Table 2 (Zhang et al., 2020).

**TABLE 1:** The components of Qian Yang Yu Yin.

Components	Chinese name	Family	Amount used (g)
*Cyathula officinalis* K. C. Kuan	Chuan Niu Xi	Amaranthaceae	170
*Scrophularia ningpoensis* Hemsl	Xuan Shen	Scrophulariaceae	180
*Bidens pilosa* L.	Gui Zhen Cao	Asteraceae	110
*Cornus officinalis* Siebold and Zucc	Shan Zhu Yu	Cornaceae	108
*Alisma plantago-aquatica* subsp. orientale (Sam.) Sam	Ze Xie	Alismataceae	180
*Reynoutria multiflora* (Thunb.) moldenke	He Shou Wu	Polygonaceae	180

**TABLE 2:** Quality evaluation of Qian Yang Yu Yin.

Major constituents	Method of determination	Quality specifications
2,3,5,4-TDG	HPLC	>6 mg per 10 g QYYY
Morroniside	HPLC	Contained
Harpgide	HPLC	Contained
Ecdysterone	HPLC	Contained
Hyperin	HPLC	Contained

HPLC: high performance liquid chromatogram.

### Animals and Treatment

50 spontaneously hypertensive rats (SHR), 10 Wistar-Kyoto rats (WKY), 8 weeks old, male, weighing 200 ± 30 g, were purchased from Beijing Weitong Lihua Experimental Animal Technology Co., Ltd. After four weeks of adaptive feeding, the rats were weighed and administrated. SHR were randomly divided into five groups: model group, QYYY (low, medium and high dose) group and valsartan group. Wistar-Kyoto rats (WKY) of the same age were used as the control group. WKY were fed with normal salt diet (0.4% NaCl) while SHR were fed with high salt diet (4% NaCl) for 8 weeks to establish hypertensive nephropathy model and then be treated with QYYY or valsartan for another 8 weeks. QYYY group: QYYY granule (low dose 3.2 g/kg/d, medium dose 6.4 g/kg/d, high dose 12.8 g/kg/d) was given by gavage; valsartan group: valsartan (17 mg/kg/d) was given by gavage. This dose was 10 folds of clinic dosage according to the pharmacology experimental methodology of TCM which had obvious curative effect based on the results of the previous studies (Wang, 2020). After 8 weeks’ treatment of QYYY or valsartan, serum creatinine (S-Cr), serum cystatin C (CysC), blood urea nitrogen (BUN), urinary β2-microglobulin (β2-MG), urinary N-acetyl-β-glucosaminidase (NAG), and urinary microalbumin (mALB) were assessed. The changes of HIF-1α, GLUT1, CTGF, IL-6, TGF-β1, ATP, lactate, pyruvate, and pathology were also assessed *in vivo*. The Experimental Animal Ethics Committee of Affiliated Hospital of Nanjing University of Chinese Medicine approved all animal experiments.

### Cell Culture

HEK293T cell was purchased from Cell Bank of the Chinese Academy of Sciences (Shanghai, China) and cultured with Dulbecco’s modified Eagle’s medium (DMEM; Gibco, United States of America) containing 10% fetal bovine serum (FBS; Gibco, United States of America) in a humidified 5% CO_2_ atmosphere at 37°C.

### Preparation of Qian Yang Yu Yin-Containing Serum

Thirty 8 week-old Sprague Dawley rats (SD), male, weighing 200 ± 30 g, were purchased from Beijing Weitong Lihua Experimental Animal Technology Co., Ltd. After four weeks of adaptive feeding, the rats were weighed and administrated. SD were randomly divided into five groups: blank group (*n* = 10), positive control group (*n* = 5) and QYYY group (*n* = 15). Blank group: pure water was given with 1 ml/100 g by gavage; positive control group: valsartan was given according to 17 mg/kg/d; QYYY group: QYYY granule was given by gavage according to 12.8 g/kg/d. Each group was given drug intervention for seven consecutive days. Thereafter, the rats were anesthetized and their blood was drawn from abdominal aorta. 5–8 ml blood was collected from each rat and kept in the blood collection tube at room temperature for 2 h. After centrifugation at 3,000 rpm for 10 min, the upper serum was aspirated, and the serum of the same group was mixed, filtered with 0.22 μM filter, placed into 1.5 ml sterile centrifuge tube and stored at -20°C.

### Hypoxia Treatment

We determined the optimal anoxia time according to the expression of HIF-1α. HEK 293T cells were placed in a hypoxia cultivator containing a gaseous mixture of 5% CO_2_, 1% O_2,_ and 94% N_2_ at 37°C for durations of 0, 6, 12, 24, and 36 h respectively. Subsequently, the proteins from HEK 293T cells were extracted with RIPA lysis buffer (Beyotime, China) and subjected to western blot analysis to analyze HIF-1a expression. We considered the time with the highest HIF-1α expression as our optimal anoxic exposure time. In our study, we found that HIF-1α expression peaked at 12 h of hypoxia, so we performed our experiments at 12 h of hypoxia in this study. Hypoxia was induced using a three-gas hypoxia incubator chamber (5% CO_2_, 1% O_2,_ and 94% N_2_). For drug intervention, cultured HEK 293T cells were pre-treated with QYYY for 1 h before hypoxia. Cells were randomly divided into following groups: Model group (Hypoxia + 10% blank serum); QYYY group (Hypoxia + 5% QYYY-containing serum + 5% blank serum) and valsartan group (Hypoxia + 10% Valsartan-containing serum). The control group was maintained in a humidified atmosphere of 5% CO_2_ at 37°C till end of study.

### MTT Assay

The proliferation abilities of HEK 293T cells for 0, 6, 12, 24, and 36 h in the anoxic incubator were assessed by MTT assay. HEK293T cells were cultured in 96-well plates with 10% FBS in a three-gas hypoxia incubator chamber (5% CO_2_, 1% O_2,_ and 94% N_2_) for 0, 6, 12, 24, and 36 h, respectively. Then 20 µl MTT was added and then cultured for another 4 h. Finally, 150 µl DMSO was added. Plates were shaken for 10 min at room temperature. We used microplate reader to measure absorbance at 570 nm.

### Western Blot

The protein was extracted by RIPA lysis buffer (Beyotime Biotechnology, Shanghai, China) mixed with phenylmethanesulfonyl fluoride (PMSF). The quantity of proteins was measured using the BCA assay (Beyotime Biotechnology, Shanghai, China). 10% sodium dodecyl sulfate polyacrylamide gel electrophoresis were used to separate different molecular weight proteins. Gels were then transferred onto PVDF membranes. Nonfat milk was used of for 1 h then and the membranes were incubated with the primary antibody against HIF-1α, PKM2, GLUT1, LDH-A, IL-6, and GAPDH at 4°C overnight. Peroxidase-conjugated second antibody were uesd for 1 h on the shaking table at room temperature. Protein bands were detected by ChemiDoc XRS imaging system (Bio-Rad, United States of America) after using ECL reagents.

### Real-Time Quantitative PCR Analysis

The total RNA was extracted by using Trizol (Ambion, United States). The High Capacity cDNA Reverse Transcription Kit (Vazyme, Nanjing, China) was used to conduct the reverse transcription. SYBR Green chemistry (Vazyme, Nanjing, China) on a 7,500 fast RT-PCR system was used to perform amplified reaction. The primers (Invitrogen Co, Shanghai, China) were listed in Supplementary Table S1. The ratio of the mRNA expression of the target gene vs that of *β*-actin was defined as 2^−△△Ct^.

### Lentiviral Transduction to Establish Stable Cell Lines

HIF-1α-overexpression, PKM2-overexpression, HIF-1α-knockdown, and PKM2-knockdown lentiviruses were generated by Genechem Co.,Ltd (Shanghai, China). Thereafter, over-expression and silencing lentiviral vectors of HIF-1α were transfected into 293T cells when multiplicity of infection was 10. Green fluorescent protein was then expressed in all the lentiviral vectors. We also collected other lentiviral vectors without carrying HIF-1α or PKM2 for excluding the influence of lentivirus itself on transfection. The stable cell lines were selected by using 2 μg/ml puromycin (VWR, America) in medium. The levels of HIF-1α and PKM2 were detected by using qPCR and western blotting.

### Kits for ATP, Lactate and Pyruvate

Levels of ATP, lactate and pyruvate were measured using an the assay kits (Nanjing Jiancheng Bioengineering Institute, Nanjing, China) according to the instruction of manufacturer.

### HE Staining

The kidney tissues were fixed with 4% paraformaldehyde for 24 h, dehydrated gradiently in turn, and embedded in the paraffin wax blocks. The blocks were labelled based on the different treatment groups. The wax block was thereafter sliced, dewaxed and washed. Hematoxylin staining and eosin staining were used in a sequence.

### Masson Staining

The kidney tissues were fixed and sectioned as described above under HE staining. Weigert’s iron hematoxylin staining was used to stain the nucleus, and ponceau S was used thereafter for 5–10 min. After rinsing with distilled water, they were treated with molybdophosphoric acid for 3–5 min. The samples were then stained using Aniline blue again for 5 min and treated with 1% glacial acetic acid for 1 min. The slides were then dried, mounted. The samples were finally observed by microscope and the images were analyzed.

### Immunofluorescence Staining

For immunocytochemistry, cells were seeded on poly-D-lysine precoated cell climbing films (Shanghai wohong Biotechnology Co., Ltd) in 12-well culture plate. The medium was removed and the cells were washed with PBS three times for 5 min each and then fixed with 4% paraformaldehyde for 15 min at room temperature. After washing with PBS three times for 5 min each, the fixed cells were blocked by using 0.3% Triton X-100/PBS for 1 h. Cells were incubated with the primary antibody, HIF-1α (Proteintech: 20960-1-AP), PKM2 (Proteintech: 60268-1-Ig), GLUT-1 (Proteintech: 66290-1-Ig), LDH-A (Proteintech: 19987-1-AP) diluted in 1% BSA in PBS overnight at 4°C. After washing three times with PBS, cells were incubated for 1.5 h with CoraLite488-conjugated Affinipure Goat Anti-Rabbit IgG (H + L) (Proteintech: SA00013-2) or CoraLite594-conjugated Goat Anti-Mouse IgG (H + L) (Proteintech: SA00013-3) secondary antibodies. The unbound secondary antibody was removed with three washes of PBS for 5 min each. Next, the samples were counterstained with DAPI (Beyotime Biotechnology). Samples were visualized on fluorescence microscopes (Nikon, Japan). The intensity was quantified using ImageJ software.

### Immunohistochemistry

The rat kidney tissues were fixed and processed as described above for HE staining. The tissue sections were then placed in EDTA antigen repair buffer (pH = 9.0) for 15 min. After cooling, the slides were placed in PBS (pH = 7.4). The slides were shaken and washed for three times on the shaking table for 5 min each time. The slides were then placed in 3% hydrogen peroxide solution and incubated for 25 min at room temperature in the dark. The slides were shaken and washed for three times as before. The tissues were incubated with the primary antibody overnight at 4°C in a wet box. After washing three times with PBS, the tissues were incubated with secondary antibodies for 50 min at room temperature. The secondary antibody was removed by washing three times with PBS for 5 min each. After the slides have dried, DAB solution was added to observe the color, and then stained with hematoxylin for 3 min. Thereafter, dehydration was carried out, and the slides were observed under microscope, and the images were collected and analyzed. The optical density of immunohistochemical images was analyzed by ImageJ software.

### Bioinformatics Analysis and Network Pharmacology of Qian Yang Yu Yin

All of the compound data of the six botanical drugs contained in QYYY were retrieved from TCMSP and Chemistry database, and their corresponding ADME indices, including oral bioavailability (OB) and drug likeness (DL), were collected. The screening conditions were OB ≥ 30% and DL ≥ 0.18. The putative target proteins of these active compounds were searched through TCMSP. The putative target proteins names were converted into gene names through UniProt database. With the help of Cytoscape 3.7.0 software, a network was constructed to connect the candidate compounds of QYYY with the putative targets. All of the hypertensive nephropathy-associated genes were obtained from the OMIM and GeneCards databases. The acquired genes were overlapped with the putative target proteins from QYYY. Upload the intersection target genes to the STRING platform to perform KEGG enrichment analysis and structure protein-protein interaction network.

### Statistical Analysis

SPSS 20 software was used to statistical analysis. The differences were considered statistically significant when *p* < 0.05.

## Results

### Qian Yang Yu Yin Promoted Blood Pressure, Pathology, Fibrosis, Renal Functions *In Vivo*

**FIGURE 1:**
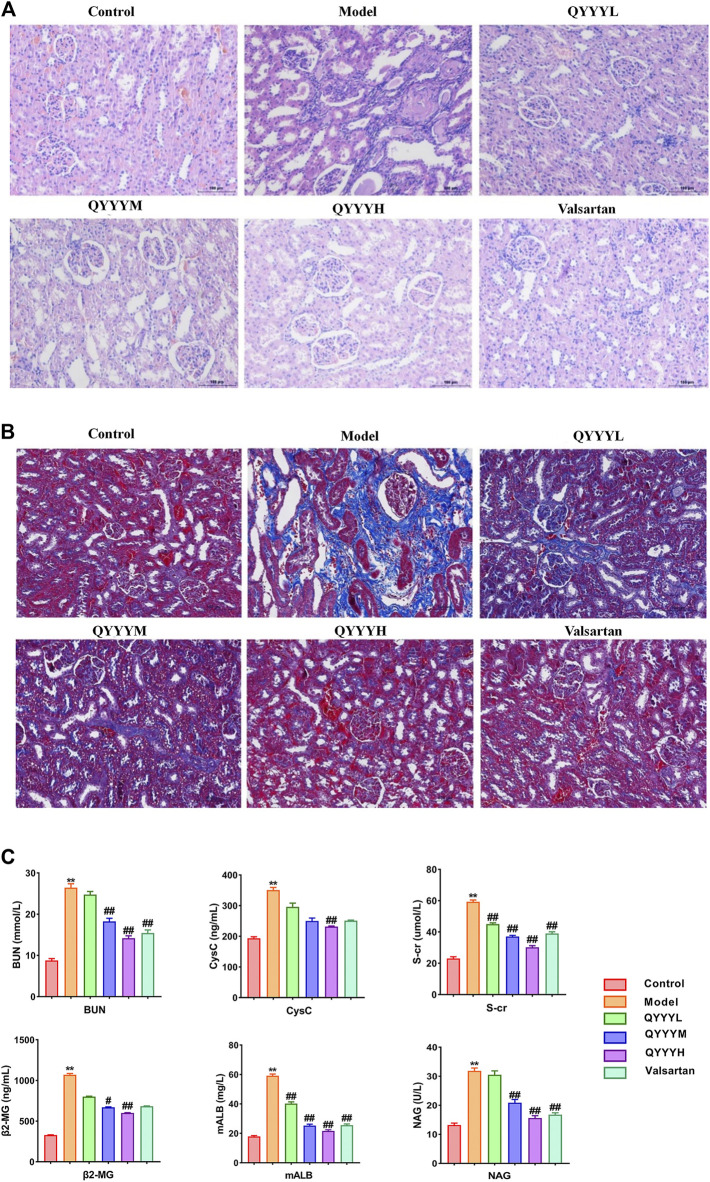
QYYY promoted pathology, fibrosis and renal function *in vivo*. SHR were randomly divided into five groups: Model group, QYYYL (low dose 3.2 g/kg/d), QYYYM (middle dose 6.4 g/kg/d), QYYYH (high dose 12.8 g/kg/d) group and valsartan group (17 mg/kg/d). WKY of the same age were used as the control group. WKY were fed with normal salt diet (0.4% NaCl) while SHR were fed with high salt diet (4% NaCl) for 8 weeks to establish hypertensive nephropathy model and then be treated with QYYY or valsartan for another 8 weeks. **(A)** Effects of QYYY on renal histomorphology in rats. **(B)** Effects of QYYY on masson staining method for staining renal fibrosis. **(C)** Effects of QYYY on renal function in SHR. * *p* < 0.05 and ** *p* < 0.01 as compared with the control group. ^#^ *p* < 0.05 and ^##^ *p* < 0.01 as compared with the model group.

Firstly, we successfully established the model by 12-week-old SHR which were fed with high salt diet (4% NaCl) for 8 weeks. We observed the effects of QYYY on BP, pathology, fibrosis and renal function *in vivo* to identify the effects of QYYY on hypertensive nephropathy. Effects of QYYY on renal histomorphology in rats were shown in Figure 1A. In model group, the arrangement of renal tubules was observed to be loose and the volume of epithelial cells was relatively smaller. In QYYYH and valsartan group, the atrophy of glomeruli was not obvious and the vacuoles in renal small cysts were improved (Figure 1A). After observing the effects of QYYY on masson staining method for staining renal fibrosis, it was found QYYY and valsartan effectively improved the renal fibrosis (Figure 1B). The effects of QYYY on indicators of BP and renal function including S-Cr, CysC, BUN, β2-MG, NAG, and mALB in rats were shown in Supplementary Tables S2 and S3; Figure 1C. The resluts showed that QYYY significantly decreased the levels of S-cr, CysC, BUN, mALB, NAG, and β2-MG in SHR. These data suggest that QYYY can treat hypertensive nephropathy effectively.

### Network Pharmacology Prediction of the Potential Active Compounds in Qian Yang Yu Yin and Corresponding Pathways Related to Hypertensive Nephropathy

**FIGURE 2:**
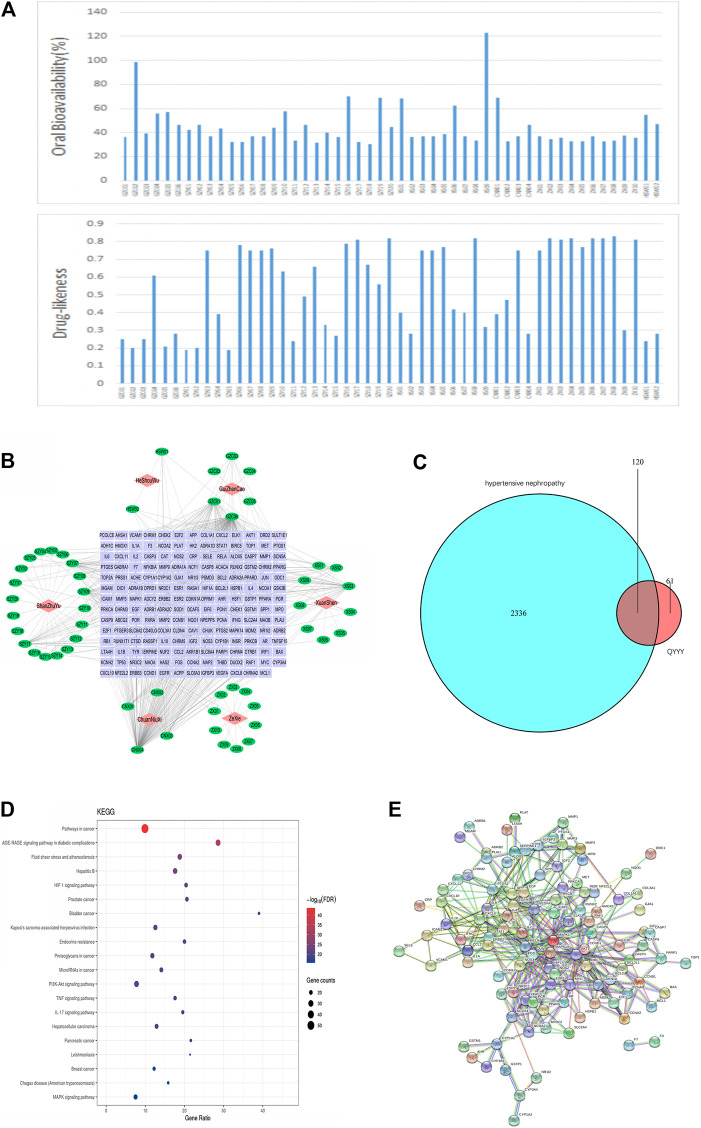
Network pharmacology prediction of the potential active compounds in QYYY and corresponding pathways related to hypertensive nephropathy. **(A)** Active compounds in QYYY with oral bioavailability (OB) ≥ 30% and drug-likeness (DL) ≥ 0.18. **(B)** Drug–Compound–Target Network: the nodes represent drug (red diamond), candidate compounds (green ellipse), and the targets proteins (purple rectangle). **(C)** Venn diagram of 2,456 hypertension nephropathy-associated genes and 181 putative target proteins screened from candidate compounds of QYYY. **(D)** KEGG analysis of the 120 gene targets. **(E)** PPI network of the 120 gene targets.

After identifying that QYYY can effectively treat hypertensive nephropathy, we performed a network pharmacology investigation of QYYY to determine its candidate active compounds and the corresponding pathways underlying the progression of hypertensive nephropathy. Firstly, a library for active compounds of the six botanical drugs in QYYY was constructed based on online databases and literature mining. Only compounds with favorable pharmacokinetic parameters of oral bioavailability (OB ≥ 30%) and drug-likeness (DL ≥ 0.18) were included (Figure 2A) and their chemical information are listed (Supplementary Table S4). Secondly, we screened 181 putative target proteins from the 46 active compounds in QYYY and 30 candidate compounds were identified. To elucidate the multiple interactions of these target proteins, we constructed a Drug–Compound–Target (D–C–T) network (Figure 2B). To further identify the effects of QYYY on hypertensive nephropathy, 2,456 hypertensive nephropathy-associated genes were extracted from databases and 120 of these genes were overlapped with putative target proteins of QYYY (Figure 2C). KEGG analysis of the 120 gene targets (Figure 2D) showed that pathways involved in HIF-1 signaling pathway. PPI network (Figure 2E) was established to understand the possible regulatory mechanism and its relationship.

### Qian Yang Yu Yin Promoted HIF-1α, PMK2, Metabolic Markers, Renal Inflammation and Fibrosis *In Vivo*

**FIGURE 3:**
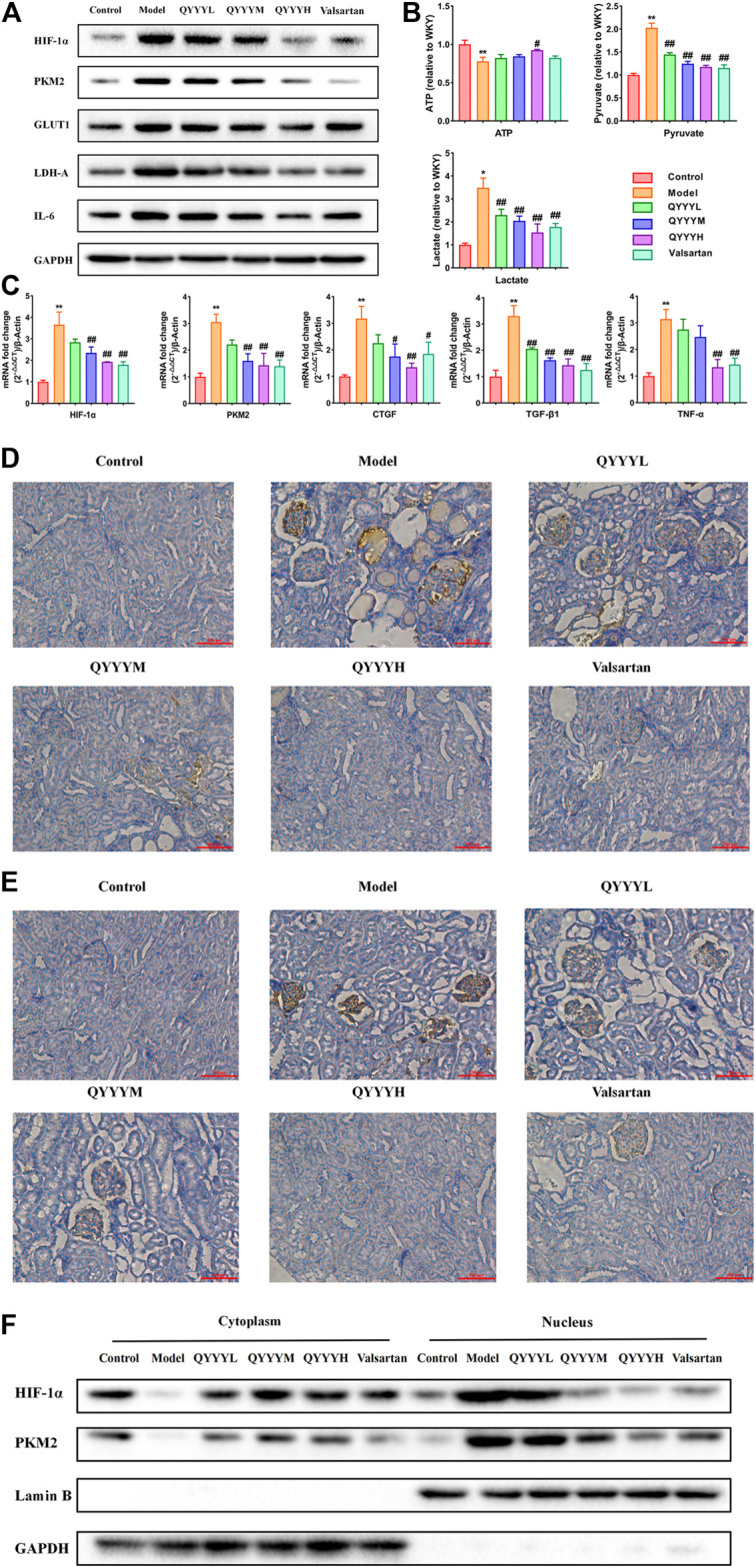
QYYY promoted HIF-1α, PKM2, metabolic markers, renal inflammation and fibrosis *in vivo*. **(A)** Effects of QYYY on protein expression of HIF-1α, PKM2, GLUT1, LDH-A, and IL-6 in SHR were examined using western blotting. **(B)** Effects of QYYY on changes of ATP, pyruvate and lactate using kits. **(C)** Effects of QYYY on mRNA levels of HIF-1α, PKM2, CTGF, TGF-β1 and TNF-α in SHR were examined using qPCR. **(D)** Effects of QYYY on protein level of HIF-1α in SHR were examined by immunohistochemistry. **(E)** Effects of QYYY on protein level of PKM2 in SHR were examined by immunohistochemistry. **(F)** Effects of QYYY on nuclear and cytoplasmic protein expression of HIF-1α, PKM2 in SHR were examined using western blotting. * *p* < 0.05 and ** *p* < 0.01 as compared with the control group. ^#^ *p* < 0.05 and ^##^ *p* < 0.01 as compared with the model group.

According to the results of the above network pharmacology prediction and some related studies. We speculated that QYYY can improve HIF-1α, PKM2 and related metabolism, inflammation, and fibrosis indexes. Therefore, we observed the effects of QYYY on HIF-1α, PMK2, metabolic markers, renal inflammation, and fibrosis *in vivo*. Western blot results indicated that QYYY significantly decreased the expression of HIF-1α, PKM2, GLUT1, LDH-A, and IL-6 in SHR (Figure 3A). The nucleoprotein expression of HIF-1α, PKM2 were significantly increased and cytoplasmic protein of HIF-1α, PKM2 were significantly decreased in model group. It triggered the nuclear translocation and increased the transcriptional activity of HIF-1α and PKM2 in model group. QYYY significantly increased the cytoplasmic level of HIF-1α compared to model group and blocked HIF-1α nuclear accumulation significantly (Figure 3F). The resluts by using kits for ATP, lactate and pyruvate indicated that QYYY significantly increased the production of ATP and decreased the production of pyruvate and lactate in SHR (Figure 3B). QPCR results showed that QYYY significantly decreased the expression of HIF-1α, PKM2, CTGF, TGF-β1, and TNF-α in SHR (Figure 3C). Immunohistochemistry showed that QYYY significantly decreased the expression of HIF-1α and PKM2 in SHR (Figures 3D,E). These data suggest that QYYY can improve metabolic markers, renal inflammation and fibrosis *in vivo* to treat hypertensive nephropathy.

### Qian Yang Yu Yin Promoted HIF-1α, PMK2, Metabolic Markers, Renal Inflammation and Fibrosis *In Vitro*

**FIGURE 4:**
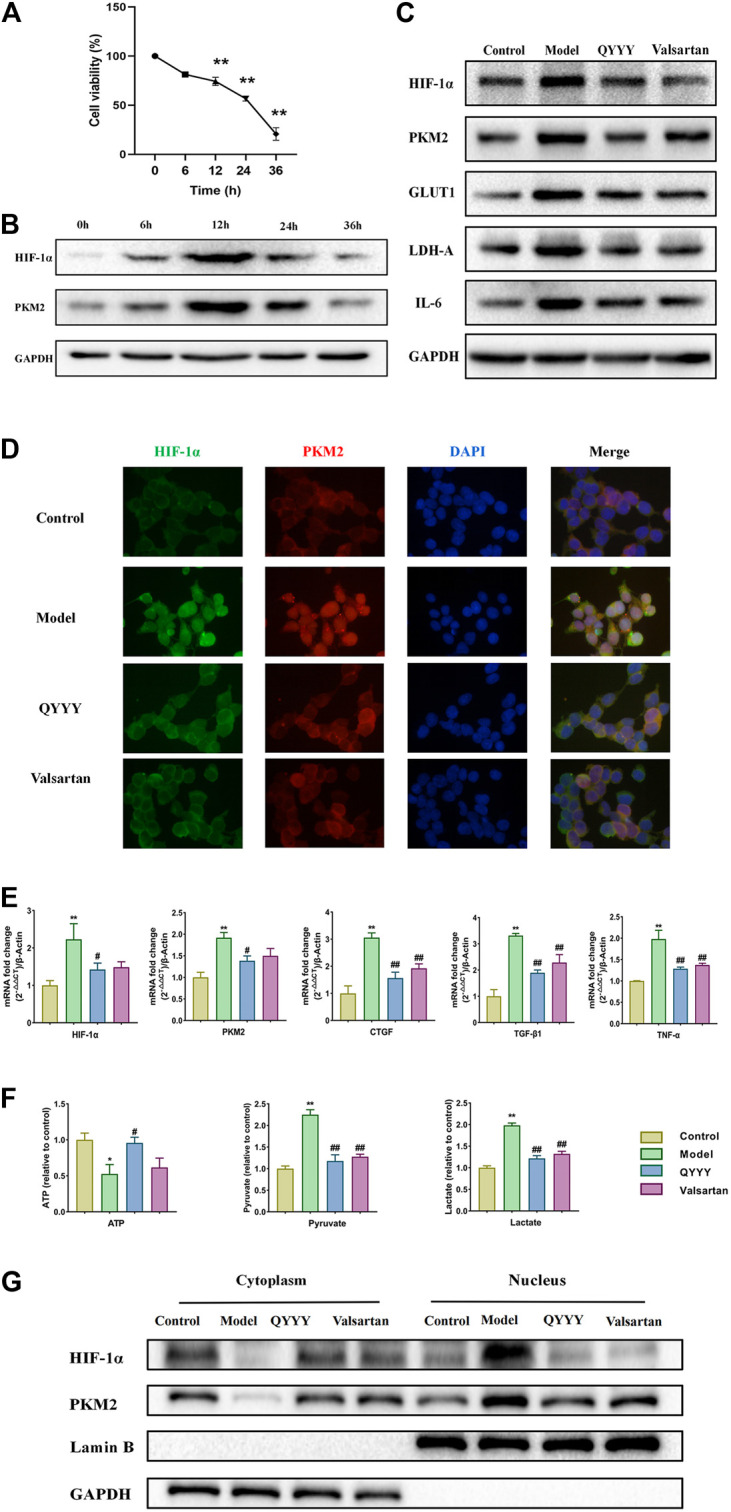
QYYY promoted HIF-1α, PMK2, metabolic markers, renal inflammation and fibrosis *in vitro*. Cells were divided into following groups: Model group (Hypoxia + 10% blank serum); QYYY group (Hypoxia + 5% QYYY-containing serum + 5% blank serum) and valsartan group (Hypoxia + 10% Valsartan-containing serum). The control group was maintained in a humidified atmosphere of 5% CO_2_ at 37°C till end of study. **(A)** Effects of anoxia time on the cell viability of HEK293T were examined by using MTT. **(B)** Effects of anoxia time on the protein expression of HIF-1α and PKM2 of hypoxic HEK293T cells were examined by using western blotting. **(C)** Effects of QYYY on protein expression of HIF-1α, PKM2, GLUT1, LDH-A, and IL-6 in hypoxic HEK293T cells were examined by using western blotting. **(D)** Effects of QYYY on immunofluorescence staining of HIF-1α and PKM2 in hypoxic HEK293T cells. **(E)** Effects of QYYY on mRNA levels of HIF-1α, PKM2, CTGF, TGF-β1, and TNF-α in hypoxic HEK293T cells were examined by using qPCR. **(F)** Effects of QYYY on changes of ATP, pyruvate and lactate using kits. **(G)** Effects of QYYY on nuclear and cytoplasmic protein expression of HIF-1α and PKM2 in hypoxic HEK293T cells were examined by using western blotting. * *p* < 0.05 and ** *p* < 0.01 as compared with the control group. ^#^ *p* < 0.05 and ^##^ *p* < 0.01 as compared with the model group.

To further determine the effects of QYYY on HIF-1α, PMK2, metabolic markers, renal inflammation, and fibrosis in hypertensive nephropathy. We also observed the curative effect *in vitro*. Hypoxia can stimulate the proliferation of mesangial and epithelial cells, thereby modulating the glomerular hemodynamics and leading to glomerular sclerosis and stimulation of tubular epithelial cells and renal interstitial fibroblasts to synthesize a large number of extracellular matrix components and reduce their degradation. HEK293T cells, human renal epithelial cells, was chosen to be the model of renal damage of the hypertension in the current study. The effects of different anoxia time on the viability of HEK293T cells were detected via MTT assay. We found there was no significant change in HEK293T cell viability at 6 h, but the cell viability was significantly decreased when treated for 12, 24, and 36 h (Figure 4A). We investigated the effect of anoxia time on HEK293T cells, which were exposed to the incremental durations of anoxia time of 0, 6, 12, 24, 36 h. The results showed that HIF-1α and PKM2 reached its peak level at 12 h of anoxia which suggested a steady anoxic condition (Figure 4B). Hence, we selected 12 h as an optimal anoxia time-point in the following study. QPCR and western blot results indicated that QYYY significantly decreased the expression of HIF-1α, PKM2, GLUT1, LDH-A, CTGF, TGF-β1, TNF-α, and IL-6 in hypoxic HEK293T cells (Figures 4C,E).The nucleoprotein expression of HIF-1α, PKM2 were significantly increased and cytoplasmic protein of HIF-1α, PKM2 were significantly decreased in model group. Hypoxia also triggered the nuclear translocation and increased the transcriptional activity of HIF-1α and PKM2 *in vitro*. The cytoplasmic level of HIF-1α were significantly increased compared to model group and HIF-1α nuclear accumulation was significantly blocked by using QYYY (Figures 4D,G). The resluts by using kits for ATP, lactate and pyruvate indicated that QYYY significantly increased the production of ATP and decreased the production of pyruvate and lactate *in vitro* (Figure 4F). These data suggest that QYYY can also improve HIF-1α, PKM2, metabolic markers, renal inflammation and fibrosis *in vitro*.

### Effects of Over-Expression and Knockdown of HIF-1α on Relative Markers in HEK293T Cells

**FIGURE 5:**
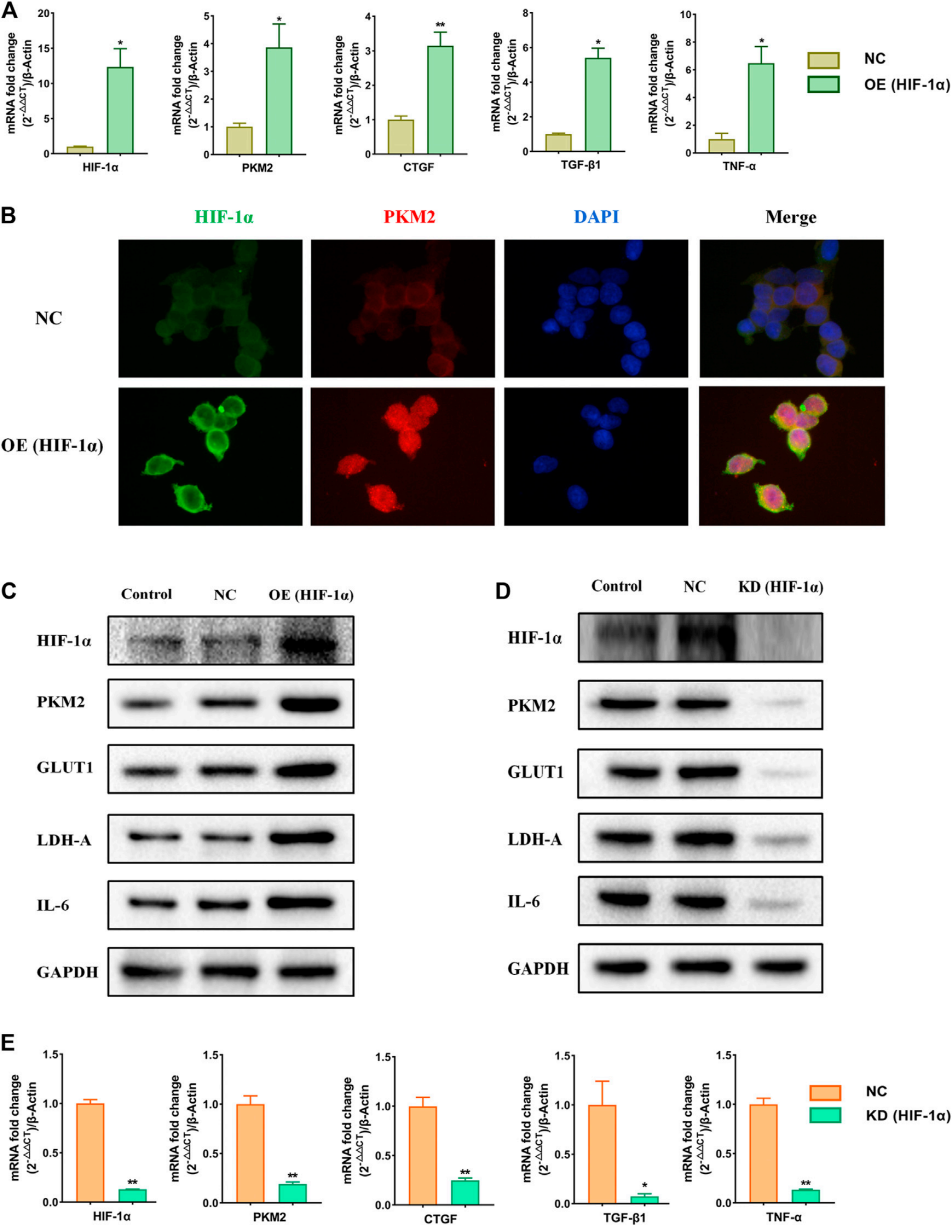
Effects of over-expression and knockdown of HIF-1α on relative markers in HEK293T cells. Cells were divided into following groups: conrtol, negative control (NC), HIF-1α-overexpression group (OE) and HIF-1α-knockdown group (KD). **(A)** Effects of over-expression of HIF-1α on mRNA levels of HIF-1α, PKM2, CTGF, TGF-β1, and TNF-α were examined by using qPCR. **(B)** Effects of over-expression of HIF-1α on immunofluorescence staining of HIF-1α and PKM2. **(C)** Effects of over-expression of HIF-1α on proteins of HIF-1α, PKM2, GLUT1, LDH-A, and IL6 in HEK293T cells were examined by using western blotting. **(D)** Effects of knockdown of HIF-1α on proteins of HIF-1α, PKM2, GLUT1, LDH-A, and IL6 in HEK293T cells were examined by using western blotting. **(E)** Effects of knockdown of HIF-1α on mRNA levels of HIF-1α, PKM2, CTGF, TGF-β1, and TNF-α in HEK293T cells were examined by using qPCR. * *p* < 0.05 and ** *p* < 0.01.

To further study the role of HIF-1α in the metabolic reprogramming, HIF-1α-overexpression and HIF-1α-knockdown lentiviruses were constructed (Supplementary Figure S1). We analyzed the changes of the expression of HIF-1α, PKM2, GLUT1, LDH-A, CTGF, TGF-β1, TNF-α, and IL-6 when HIF-1α was over expressed or knockdowned respectively. The results showed that the expressions of HIF-1α, PKM2, GLUT1, LDH-A, CTGF, TGF-β1, TNF-α, and IL-6 were increased significantly following the HIF-1α over-expression (Figures 5A–C). Over-expression of HIF-1α triggered the nuclear translocation and increased the transcriptional activity of PKM2 (Figure 5B). After knockdowning HIF-1α, the expression of HIF-1α, PKM2, GLUT1, LDH-A, CTGF, TGF-β1, TNF-α, and IL-6 was significantly decreased (Figures 5D,E). The results suggest that HIF-1α is the up-stream in the metabolic reprogramming and plays a very important role in hypertensive nephropathy.

### Effects of Over-Expression and Knockdown of PKM2 on Relative Markers in HEK293T Cells

**FIGURE 6:**
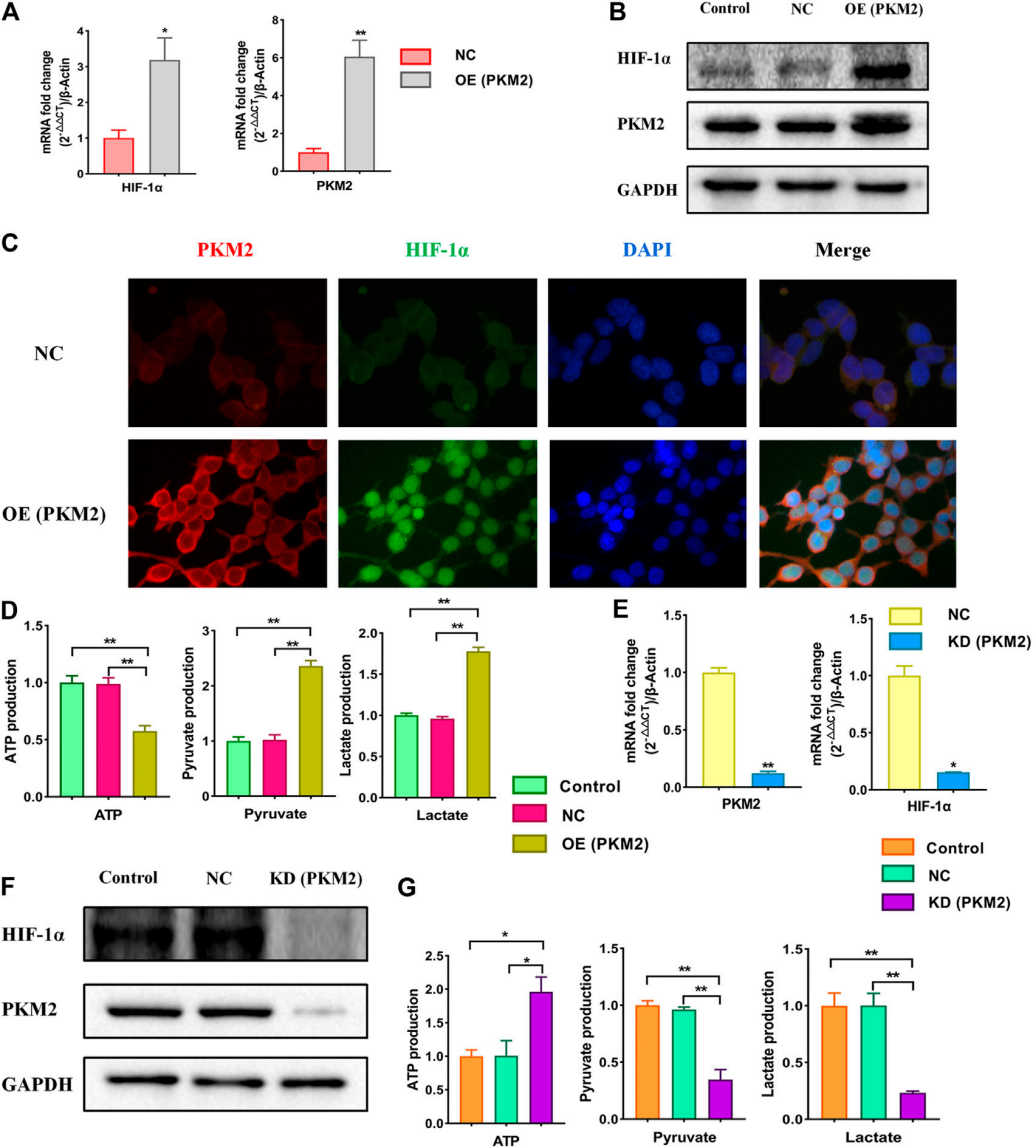
Effects of over-expression and knockdown of PKM2 on relative markers in HEK293T cells. Cells were divided into following groups: conrtol, negative control (NC), PKM2-overexpression group (OE) and PKM2-knockdown group (KD). **(A)** Effects of over-expression of PKM2 on mRNA levels of HIF-1α and PKM2 were examined by using qPCR. **(B)** Effects of over-expression of PKM2 on proteins of HIF-1α and PKM2 in HEK293T cells were examined by using western blotting. **(C)** Effects of over-expression of PKM2 on immunofluorescence staining of HIF-1α and PKM2. **(D)** Effects of over-expression of PKM2 on changes of ATP, pyruvate and lactate using kits. **(E)** Effects of knockdown of PKM2 on mRNA levels of HIF-1α and PKM2 in HEK293T cells were examined by using qPCR. **(F)** Effects of knockdown of PKM2 on proteins of HIF-1α and PKM2 in HEK293T cells were examined by using western blotting. **(G)** Effects of knockdown of PKM2 on changes of ATP, pyruvate and lactate using kits. * *p* < 0.05 and ** *p* < 0.01.

A large number of studies have confirmed that HIF-1α is closely related to PKM2 and there is a strong mutual influence between them. Therefore, we speculated that HIF-1α/PKM2 positive feedback is the key pathway of metabolic reprogramming of renal cell in hypertension (Luo et al., 2011). As is shown above, the results showed that the expressions of PKM2 was increased significantly following the HIF-1α over-expression. After knockdowning HIF-1α, the expression of PKM2 was significantly decreased. To further study the influence of PKM2 on HIF-1α and the role of PKM2 in the metabolic reprogramming, PKM2-overexpression and PKM2-knockdown lentiviruses were constructed (Supplementary Figure S2). We analyzed the changes of the expression of HIF-1α, PKM2, ATP, pyruvate and lactate when PKM2 was over expressed or knockdowned respectively. The results showed that the expressions of HIF-1α, PKM2, pyruvate and lactate were increased and ATP was decreased significantly following the PKM2 over-expression (Figures 6A–D). Over-expression of PKM2 triggered the nuclear translocation and increased the transcriptional activity of HIF-1α (Figure 6C). After knockdowning PKM2, the expressions of HIF-1α, PKM2, pyruvate, and lactate were significantly decreased and ATP was significantly increased (Figures 6E–G). The results suggest that the relationship of HIF-1α and PKM2 was positive feedback.

### Rescue Assays Certify HIF-1α as a Target of Qian Yang Yu Yin in Hypertensive Nephropathy

**FIGURE 7:**
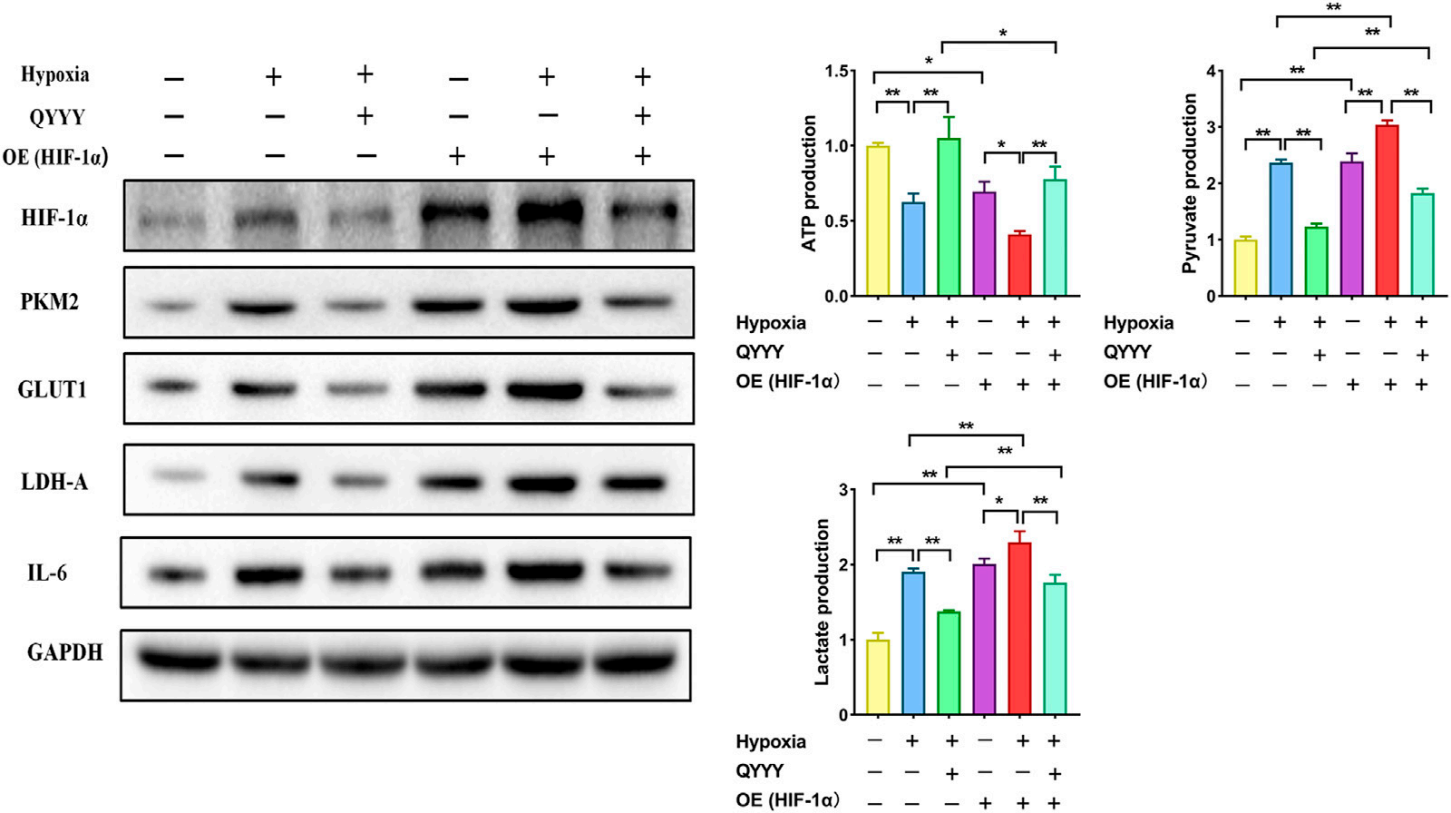
Rescue assays certify HIF-1α as a target of QYYY in hypertensive nephropathy. Over-expression of HIF-1α partially abolished the effects of QYYY on hypertensive nephropathy. * *p* < 0.05 and ** *p* < 0.01.

As is shown above, the relationship of HIF-1α and PKM2 was positive feedback. To further verify that HIF-1α was a target of QYYY in hypertensive nephropathy and study the role of HIF-1α and PKM2 in the metabolic reprogramming, we observed the effect of QYYY on HIF-1α and its downstream PKM2, GLUT1, LDH-A, and IL-6 in hypoxic HEK 293T cells in which HIF-1α was over expressed and found that over-expression of HIF-1α partially abolished the effects of QYYY on hypertensive nephropathy. The results showed that the protein expressions of HIF-1α, PKM2, GLUT1, LDH-A, IL-6, and levels of pyruvate and lactate in Hypoxia + QYYY were significantly lower than those in HIF-1α-OE + Hypoxia + QYYY. The level of ATP in Hypoxia + QYYY were significantly higher than those in HIF-1α-OE + Hypoxia + QYYY (Figure 7). These data suggest that HIF-1α is a target of QYYY and HIF-1α/PKM2 positive feedback palys an important role in improving renal injury of hypertension by regulating metabolic reprogramming.

## Disscussion

Our experimental data demonstrated that QYYY improved renal injury of hypertension by regulating metabolic reprogramming mediated by HIF-1α/PKM2 positive feedback *in vivo* and *in vitro*.

In TCM, hypertension mostly belongs to “vertigo” and “headache” category. Based on clinical and epidemiological studies of TCM, QYYY has been reported to exert the effect of tonifying the liver and kidney, promoting blood circulation and dredging collaterals. It has been awarded the national invention patent (Patent No: ZL 201010205024.2) and the Preparation Certificate of Jiangsu food and drug administration. Clinical research showed that QYYY significantly improved the early renal damage of hypertension. QYYY contains six Chinese medical botanical drugs. HPLC was established to determine the active components in QYYY and 2,3,5,4-TDG, morroniside, harpgide, ecdysterone, and hyperin were detected (Zhang et al., 2020). In recent years, a number of previous studies have reported that 2,3,5,4-TDG can protect the kidney by reducing the expression of TGF-β1 mRNA (Li et al., 2010; Stanton, 2011). 2,3,5,4-TDG is also closely related to HIF-1 and a related study (Yang and Liu, 2016) has found that it can significantly reduce the production of HIF-1, thus delaying the progression of the disease. Morroniside also has been reported to be useful in protecting the kidney (Liu et al., 2018; Jin et al., 2019). Moreover, disorder of glucose metabolism is still a difficult problem in medical field. Ecdysterone can significantly enhance glucose metabolism, improve the excretion of urinary albumin, inhibit the expression of CTGF and collagen Ⅳ, and has the potential effect of improving renal fibrosis. It acts as a potential fibrosis antagonist for the renal proximal tubule cells. It might also act through suppressing post-receptor signaling of TGF-β1 and restoring the tubule epithelial character (Zou et al., 2010; Hung et al., 2012; Chen et al., 2017). Hyperin can inhibit renal fibrosis in the rats effectively. It can reduce *a*-smooth muscle actin and fibronectin and improve the renal fibrosis (Yan et al., 2014). Therefore, we assume that improvement of metabolic disorder and anti-fibrosis of these compounds may be involved in the inhibitory effect of QYYY on treating renal injury of hypertension. Firstly, we successfully established the model by 12-week-old SHR which were fed with high salt diet (4% NaCl) for 8 weeks and we found QYYY improved BP, pathology, fibrosis, and renal function *in vivo*. In the present study, QYYYL (low dose 3.2 g/kg/d), QYYYM (middle dose 6.4 g/kg/d) and QYYYH (high dose 12.8 g/kg/d) were used and we found that the curative effects were statistically significant when administered in low dosage *in vivo*. In recent years, more and more authoritative studies have emphasized that the metabolic rate of rodents is higher and the results may be meaningless when the starting dose of rodents is too high. The evaluation of doses that are much higher than what can be achieved in humans may have no translational value from a therapeutic point of view. The importance of reasonable initial dosage for experiment was highlighted by relevant authoritative experts (Zou et al., 2012; White and Kearney, 2014; Nair and Jacob, 2016; Heinrich et al., 2020), therefore it is very necessary for us to use a lower starting dose in future experiments. From the existing experimental results, it was identified QYYY had good effects on hypertensive nephropathy. As a TCM, the components present in QYYY are complex and there are many related targets in QYYY, so it is difficult to decipher the mechanism of actions of QYYY as a whole. In recent years, the advent of network pharmacology has provided a novel method to identify the various active components of TCM, predict its related targets, and decipher its molecular mechanisms, which could be helpful to understand the different complex interactions between the biological systems, drugs and complex diseases from the perspective of network (Li and Zhang, 2013; Boezio et al., 2017; Guo et al., 2019; Zhang et al., 2019; Zhang et al., 2019; Luo et al., 2020). So we then performed a network pharmacology investigation of QYYY to determine its candidate active compounds and the corresponding pathways underlying the progression of hypertensive nephropathy. And the results showed that HIF-1 signaling pathway is a key pathway of QYYY. Moreover, our previous study also found that QYYY improved the early renal damage of hypertension by inhibiting inflammatory reaction and reducing the expression of HIF-1α, some inflammatory factors and fibrosis indexes (Ding et al., 2015; Yan et al., 2018; Wu, 2020). Many literatures also have shown the strong mutual influence between HIF-1α and PKM2 and positive feedback of HIF-1α and PKM2. HIF-1α/PKM2 positive feedback may be involved in the regulation of hypertensive renal damage by enhancing cell glycolysis (Zhu et al., 2012; Prigione et al., 2014; Wang et al., 2014; Zhu et al., 2014; Luo et al., 2015). Therefore, we speculated that QYYY may affect hypertensive nephropathy via metabolic reprogramming mediated by HIF-1α/PKM2 positive feedback. As expected, our resluts showed QYYY significantly improved the metabolic indexes, inflammatory fibrosis factors related to HIF-1α/PKM2 positive feedback *in votro* and *vivo*.

Metabolic reprogramming refers to the change of cell metabolism, which has been extensively studied and an in-depth understanding of this process has been achieved in tumor biology and other fields (Biswas, 2015; Georgoudaki et al., 2016; Li and Zhang, 2016; Schito and Semenza, 2016; Vaupel et al., 2019; Jin et al., 2020). However, it is still in its infancy in the field of research related to the kidney diseases, some evidences have shown that metabolic reprogramming plays an important role in the development of kidney diseases. As a result, novel studies elucidating the possible role of metabolic reprogramming in hypertensive nephropathy is of great significance for the treatment of renal damage of hypertension. Kidney is a high-energy consuming organ and optimal energy metabolism system is the biochemical basis for maintaining the specific structure and physiological function of kidney. During the early stage of chronic kidney disease, the glomerulus is generally characterized by high filtration and high perfusion. The glomerulus needs more energy. Fibroblasts are often activated and can proliferate rapidly in the renal interstitium. The local area of glomerular hypertrophy represents an anoxic state. In addition, when the glomerulus is in a state of hyperfiltration, the proton pump, which plays the role of renal tubular reabsorption, can also effectively reabsorb a large number of different ions in the urine and thereby increase the consumption of ATP in kidney. Under pathological conditions, the normal oxidative phosphorylation of glucose cannot provide sufficient energy or carbon groups to enable cell proliferation, so the ratio of glycolysis pathway increases. Although the increased ratio of glycolysis pathway can provide sufficient energy and necessary materials for maintaining cell proliferation and division, a few studies have reported that glycolysis pathway can increase renal fibrosis. Additionally, Some studies have also verified the potential relationship between glycolysis and renal fibrosis both *in vivo* and *in vitro*. These reports used mice with UUO as the model of renal fibrosis and found that with an increase of the degree of renal fibrosis, the levels of glucose metabolism related enzymes in the renal tissue increased synchronously. Additionally, the findings *in vitro* experiments have suggested that TGF-β1 could potentially induce renal fibroblast fibrosis and metabolic reprogramming thus increasing glycolysis pathway (Yin et al., 2018). It has been suggested that metabolic reprogramming occurs in the process of renal fibrosis and the enhancement of glycolysis can increase renal fibrosis. Therefore, inhibition of renal glycolysis may function as a new target for the treatment of chronic kidney disease. For instance, Hui Peng found that the renal injury and fibrosis could be significantly improved by improving renal energy metabolism (Peng et al., 2017). PKM2 is a key rate-limiting enzyme involved in the process of glycolysis, which could directly regulate the occurrence of glycolysis and has been reported to be closely related to embryonic development, tissue fibrosis, tumor, and glucose metabolism (Luo et al., 2011). Our data suggested that the total protein expression of PKM2 *in vivo* and *vitro* was upregulated and nuclear translocation of PKM2 was enhanced, thereby affecting glycolysis, decreasing ATP, increasing pyruvic acid and lactic acid and aggravated inflammation and fibrosis. QYYY effectively improved hypertensive renal damage through metabolic reprogramming. Glycolysis inhibitors can be used as a potential anti fibrosis strategy. Thus, the process of metabolic reprogramming has a significant potential in the study of hypertensive renal damage.

**FIGURE 8:**
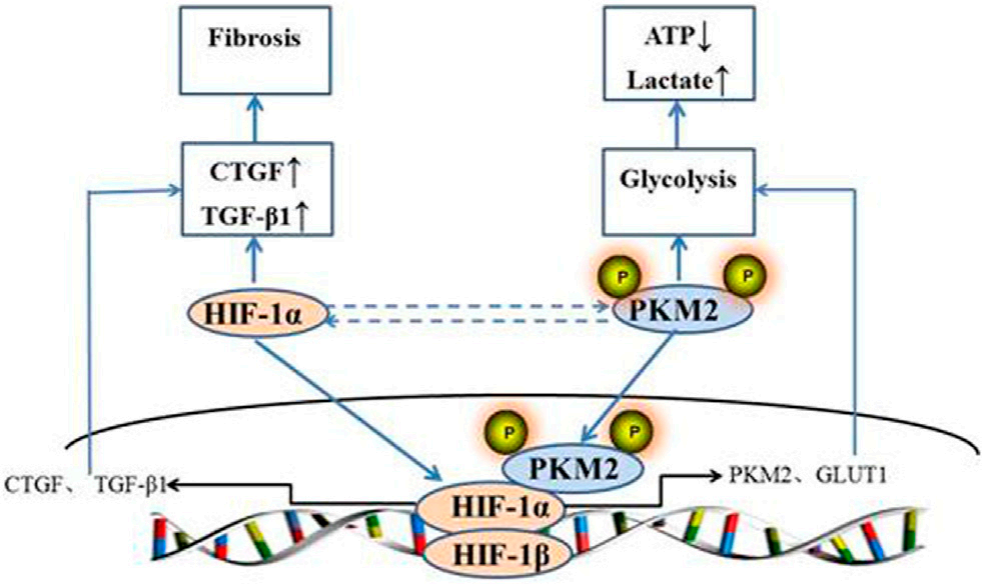
Schematic diagram of metabolic reprogramming of hypertensive kidney injury promoted by HIF-1α/PKM2 positive feedback.

HIF is a special protein discovered by Semenza in 1992 (Semenza, 1999). HIF-1α is ubiquitously found in the mammalian cells and plays a role in multiple signaling pathways (Liu et al., 2020). In recent years, the role of HIF-1α in hypertensive renal damage has attracted more and more attention. Many factors present in the disease microenvironment, such as hypoxia, reactive oxygen species, nitric oxide, and some metabolites can increase the protein level of HIF-1α and thus enhance cellular erobic glycolysis, which is a significant feature of renal injury (Gerald et al., 2004; Hsu and Sabatini, 2008; Semenza, 2013; Briggs et al., 2016; Oh et al., 2016; Magistroni and Boletta, 2017). The activation of HIF-1α signaling pathway may be involved in the regulation of hypertensive renal damage by enhancing cell glycolysis. A large number of studies have confirmed that HIF-1α is closely related to PKM2. A few studies have suggested that HIF-1α can potentially regulate reprogramming through early translocation of glycolysis and up regulation of PKM2. For instance, Luo W upregulated the expression of PKM2 through viral transfection and found that the fluorescence level of HIF-1α was significantly enhanced but the transcriptional activity of HIF-1α was significantly reduced by knockout of PKM2. Proline hydroxylase 3 can hydroxylate the proline at position 403 and 408 of PKM2, which can facilitate the binding of PKM2 with HIF-1α, and thereby promote the binding of HIF-1α with HRE, thus regulating the transcription of downstream genes such as lactate dehydrogenase and glucose transporter. The relationship between HIF and PKM is positive feedback (Prigione et al., 2014). Our data identified the role of HIF-1α and PKM2 in the metabolic reprogramming in hypertensive renal damage. QYYY played an important role in the treatment of hypertensive renal damage by regulating metabolic reprogramming mediated by HIF-1α/PKM2 positive feedback loop and HIF-1α is a key target in the treatment of renal damage of hypertension by QYYY. We found that QYYY inhibited total protein expression of HIF-1α as well as PKM2 and caused a significant increase in the accumulation of nuclear protein of HIF-1α and PKM2, thereby improving their downstream target genes by regulating metabolic reprogramming such as erobic glycolysis induced by HIF-1α and PKM2 and inhibited inflammation and fibrosis of kidney tissue. And the schematic diagram of metabolic reprogramming of hypertensive kidney injury promoted by HIF-1α/PKM2 positive feedback is shown in Figure 8.

## Conclusions

The present study demonstrated the effects of QYYY on the progress of hypertensive nephropathy and we investigated the underlying mechanisms involved in HIF-1α/PKM2 positive feedback. Our present findings clearly indicated that QYYY inhibited the positive feedback of HIF-1α and PKM2, reduced the nuclear accumulation of HIF-1α and PKM2, regulated metabolic reprogramming, and then suppressed renal inflammatory fibrosis and improved hypertensive renal injury. In total, our study provides the basis for the treatment of hypertensive renal injury with TCM by regulating metabolic reprogramming mediated by HIF-1α/PKM2 positive feedback.

## Data Availability

The original contributions presented in the study are included in the article/Supplementary Material, further inquiries can be directed to the corresponding authors.
