# Supplementary material for: Qian Yang Yu Yin Granule Improves Renal Injury of Hypertension by Regulating Metabolic Reprogramming Mediated by HIF-1α/PKM2 Positive Feedback Loop
Source: Front Pharmacol. 2021 Jun 7;12:667433. doi: 10.3389/fphar.2021.667433 (PMC8218631; doi:10.3389/fphar.2021.667433)
Supplement: Supplementary file 1 [file DataSheet1.docx]

**Supporting Information**

content

[Table S1 | Primers used in Real-Time Quantitative PCR analysis. 2](#_Toc18438)

[Table S2 | Effects of QYYY on systolic pressure (SBP) in SHR. 2](#_Toc18438)

[Table S3 | Effects of QYYY on systolic pressure (DBP) in SHR. 2](#_Toc26127)

[Table S4 | Basic information of main active components in QYYY. 3](#_Toc26127)

[Figure S1 | The results observed by fluorescence microscopy after HEK293T cells were transfected with virus (HIF-1α). 5](#_Toc26127)

[Figure S2 | The results observed by fluorescence microscopy after HEK293T cells were transfected with virus (PKM2). 5](#_Toc26127)

# Table S1 | Primers used in Real-Time Quantitative PCR analysis.

| Target Gene | Forward Primer | Reverse Primer |
| --- | --- | --- |
| Rat HIF-1α | 5’-TATCTGAAAGCCCTGGATGG-3’ | 5’-TGgTCACatggatgggtaaa-3’ |
| Rat PKM2 | 5’-cTGGACATTGACTCCGCACCC-3’ | 5’-TGCCCACCTCCACCACCTTG-3’ |
| Rat TNF-α | 5’-GGGCTCAGAATTTCCAACAA-3’ | 5’-ATCCACTCAGGCATCGACAT-3’ |
| Rat CTGF | 5’-AGAGTGGAGCGCCTGTTCTA-3’ | 5’-CCACAGAACTTAGCCCGGTA-3’ |
| Rat TGF-β1 | 5’-ACCGCAACAACGCAATCTAT-3’ | 5’-AGACAGCCACTCAGGCGTAT-3’ |
| Rat β-actin | 5’-GAGGGAAATCGTGCGTGAC-3’ | 5’-CTGGAAGGTGGACAGTGAG-3’ |
| Human HIF-1α | 5’-AGTGTACCCTAACTAGCCG-3’ | 5’-CACAAATCAGCACCAAGC-3’ |
| Human PKM2 | 5’-ATCCACGCTGGATAACGCCTAC-3’ | 5’-TGCCTTGCGGATGAATGACG-3’ |
| Human TNF-α | 5’-AGCCCATGTTGTAGCAAACC-3’ | 5’-TGAGGTACAGGCCCTCTGAT-3’ |
| Human CTGF | 5’-CTGCACCAGCATGAAGACAT-3’ | 5’-CTCCGGGACAGTTGTAATGG-3’ |
| Human TGF-β1 | 5’-CAACAATTCCTGGCGATACC-3’ | 5’-AACCCGTTGATGTCCACTTG-3’ |
| Human β-actin | 5’-GACCTGACTGACTACCTC-3’ | 5’-TCTTCATTGTGCTGGGTGC-3’ |

**Table S2 | Effects of QYYY on systolic pressure (SBP) in SHR (Data showing means ± S.E.M, mmHg )**

| Group | Number (n) | before medication | Number (n) | after 4-week medication | Number (n) | after 8-week medication |
| --- | --- | --- | --- | --- | --- | --- |
| WKY | 10 | 125.8 ± 5.8 | 10 | 126.8 ± 5.5 | 10 | 127.3 ± 6.1 |
| Model | 10 | 185.5 ± 6.1**^**^** | 8 | 187.0 ± 6.3**^**^** | 7 | 185.0 ± 2.4**^**^** |
| QYYYL | 10 | 185.3 ± 7.3**^**^** | 9 | 166.4 ± 6.8^##^ | 8 | 148.5 ± 4.8^##^ |
| QYYYM | 10 | 181.8 ± 4.3**^**^** | 10 | 161.1 ± 3.9^##^ | 8 | 137.8 ± 6.9^##^ |
| QYYYH | 10 | 190.8 ± 4.9**^**^** | 10 | 164.5 ± 4.5^##^ | 10 | 134.8 ± 6.0^##^ |
| Valsartan | 10 | 183.1 ± 8.5**^**^** | 10 | 149.6 ± 7.0^##^ | 9 | 133.6 ± 5.3^##^ |

**Table S3 | Effects of QYYY on systolic pressure (DBP) in SHR (Data showing means ± S.E.M, mmHg )**

| Group | Number (n) | before medication | Number (n) | after 4-week medication | Number (n) | after 8-week medication |
| --- | --- | --- | --- | --- | --- | --- |
| WKY | 10 | 75.9 ± 6.8 | 10 | 78.1 ± 5.5 | 10 | 76.7 ± 8.5 |
| Model | 10 | 134.6 ± 8.1**^**^** | 8 | 137.9 ± 6.4**^**^** | 7 | 139.4 ± 4.8**^**^** |
| QYYYL | 10 | 137.4 ± 7.9**^**^** | 9 | 130.6 ± 5.7^#^ | 8 | 108.1 ± 6.8^##^ |
| QYYYM | 10 | 138.5 ± 5.6**^**^** | 10 | 110.6 ± 5.6^##^ | 8 | 94.6 ± 8.5^##^ |
| QYYYH | 10 | 139.2 ± 6.0**^**^** | 10 | 100.6 ± 4.8^##^ | 10 | 87.4 ± 4.2^##^ |
| Valsartan | 10 | 138.3 ± 5.7**^**^** | 10 | 97.6 ± 7.3^##^ | 9 | 89.6 ± 4.7^##^ |

# Table S4 | Basic information of main active components in QYYY.

|  | Code | Mol ID | Molecule Name |
| --- | --- | --- | --- |
| GuiZhenCao | GZC01 | MOL000006 | luteolin |
|  | GZC02 | MOL006436 | Okanin |
|  | GZC03 | MOL006438 | (2E)-2-(3,4-dihydroxybenzylidene)-6,7-dihydroxy-benzofuran-3-one |
|  | GZC04 | MOL006441 | bidenphenol glucoside |
|  | GZC05 | MOL006442 | (R)-2-(3,4-dihydroxyphenyl)-6,7-dihydroxybenzofuran-3(2H)-one |
|  | GZC06 | MOL000098 | quercetin |
| ShanZhuYu | SZY01 | MOL001494 | Mandenol |
|  | SZY02 | MOL001495 | Ethyl linolenate |
|  | SZY03 | MOL001771 | poriferast-5-en-3beta-ol |
|  | SZY04 | MOL002879 | Diop |
|  | SZY05 | MOL002883 | Ethyl oleate (NF) |
|  | SZY06 | MOL003137 | Leucanthoside |
|  | SZY07 | MOL000358 | beta-sitosterol |
|  | SZY08 | MOL000359 | sitosterol |
|  | SZY09 | MOL000449 | Stigmasterol |
|  | SZY10 | MOL005360 | malkangunin |
|  | SZY11 | MOL005481 | 2,6,10,14,18-pentamethylicosa-2,6,10,14,18-pentaene |
|  | SZY12 | MOL005486 | 3,4-Dehydrolycopen-16-al |
|  | SZY13 | MOL005489 | 3,6-Digalloylglucose |
|  | SZY14 | MOL005503 | Cornudentanone |
|  | SZY15 | MOL005530 | Hydroxygenkwanin |
|  | SZY16 | MOL005531 | Telocinobufagin |
|  | SZY17 | MOL008457 | Tetrahydroalstonine |
|  | SZY18 | MOL000554 | gallic acid-3-O-(6'-O-galloyl)-glucoside |
|  | SZY19 | MOL005552 | gemin D |
|  | SZY20 | MOL005557 | lanosta-8,24-dien-3-ol,3-acetate |
| XuanShen | XS01 | MOL001925 | paeoniflorin_qt |
|  | XS02 | MOL002222 | sugiol |
|  | XS03 | MOL000358 | beta-sitosterol |
|  | XS04 | MOL000359 | sitosterol |
|  | XS05 | MOL007657 | scropolioside A_qt |
|  | XS06 | MOL007658 | 14-deoxy-12(R)-sulfoandrographolide |
|  | XS07 | MOL007659 | scropolioside D |
|  | XS08 | MOL007660 | scropolioside D_qt |
|  | XS09 | MOL007662 | harpagoside_qt |
| ChuanNiuXi | CNX01 | MOL012286 | Betavulgarin |
|  | CNX02 | MOL012298 | Rubrosterone |
|  | CNX03 | MOL000358 | beta-sitosterol |
|  | CNX04 | MOL000098 | quercetin |
| ZeXie | ZX01 | MOL000359 | sitosterol |
|  | ZX02 | MOL000830 | Alisol B |
|  | ZX03 | MOL000831 | Alisol B monoacetate |
|  | ZX04 | MOL000832 | alisol,b,23-acetate |
|  | ZX05 | MOL000849 | 16β-methoxyalisol B monoacetate |
|  | ZX06 | MOL000853 | alisol B |
|  | ZX07 | MOL000854 | alisol C |
|  | ZX08 | MOL000856 | alisol C monoacetate |
|  | ZX09 | MOL002464 | 1-Monolinolein |
|  | ZX10 | MOL000862 | [(1S,3R)-1-[(2R)-3,3-dimethyloxiran-2-yl]-3-[(5R,8S,9S,10S,11S,14R)-11-hydroxy-4,4,8,10,14-pentamethyl-3-oxo-1,2,5,6,7,9,11,12,15,16-decahydrocyclopenta[a]phenanthren-17-yl]butyl]acetate |
| HeShouWu | HSW01 | MOL000492 | catechin |
|  | HSW02 | MOL002268 | rhein |


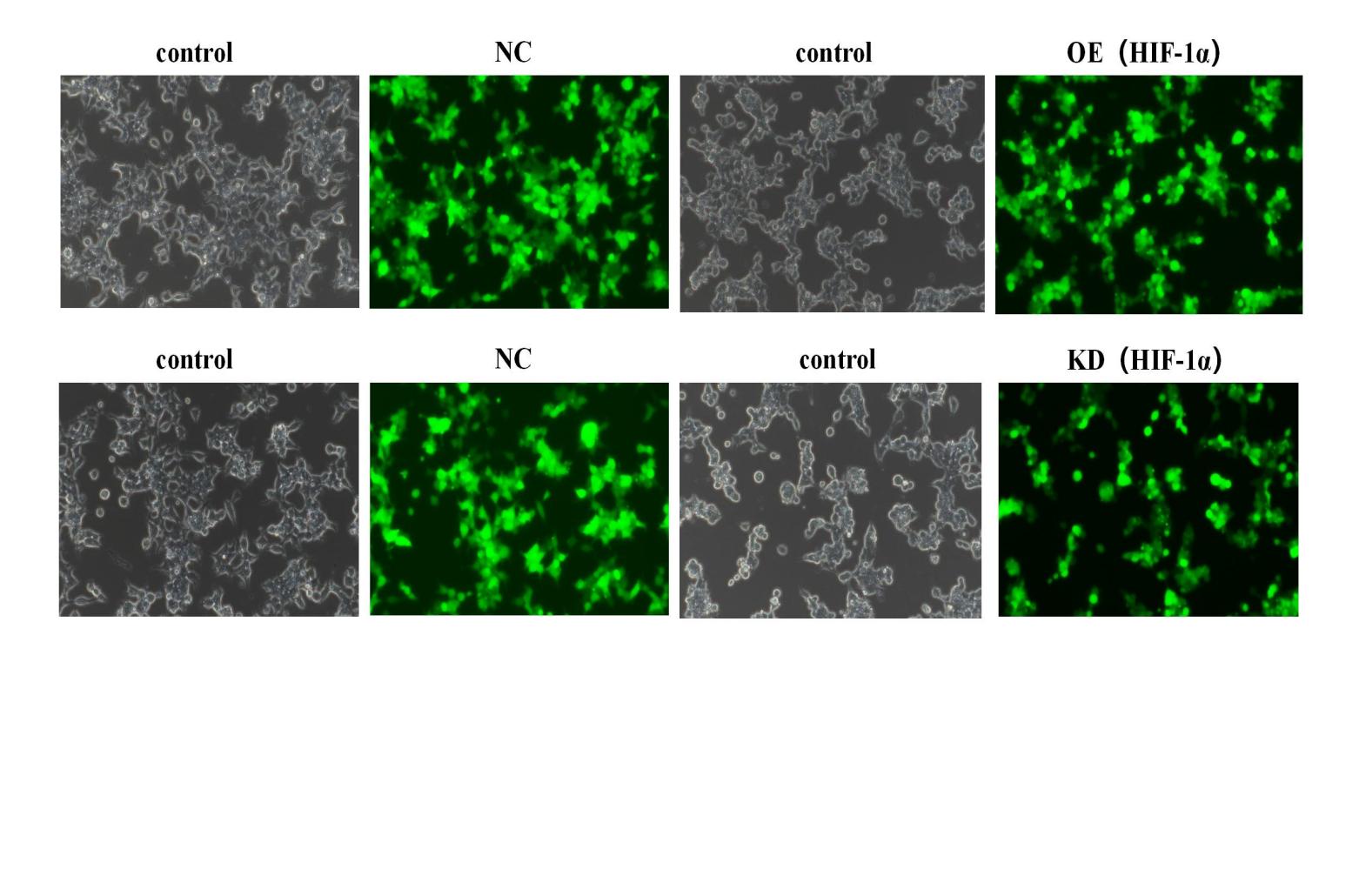


**Figure S1 | The results observed by fluorescence microscopy after HEK293T cells were transfected with virus (HIF-1α).**


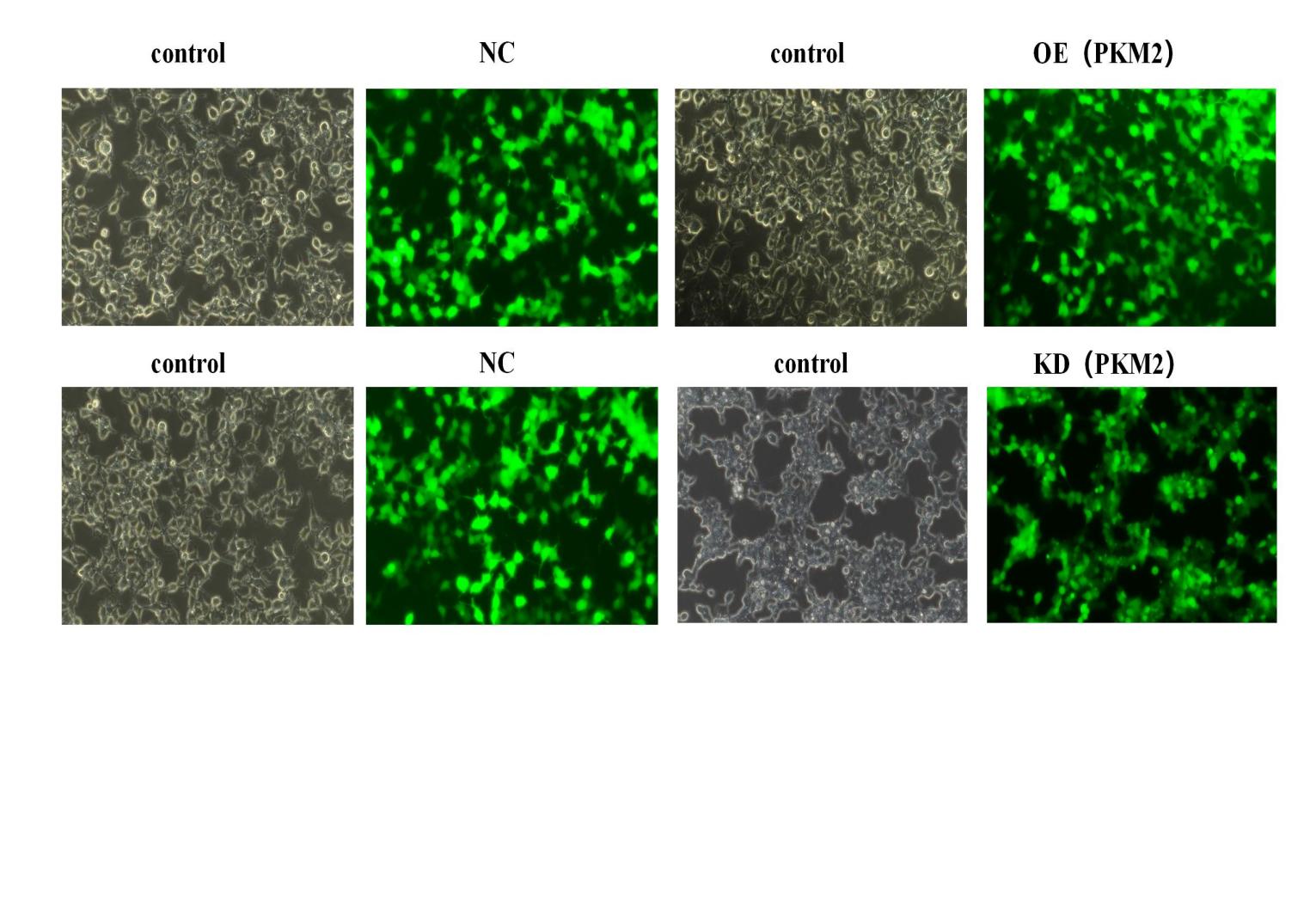


**Figure S2 | The results observed by fluorescence microscopy after HEK293T cells were transfected with virus (PKM2).**
